# Mobile Phone Use, Blood Lead Levels, and Attention Deficit Hyperactivity Symptoms in Children: A Longitudinal Study

**DOI:** 10.1371/journal.pone.0059742

**Published:** 2013-03-21

**Authors:** Yoon-Hwan Byun, Mina Ha, Ho-Jang Kwon, Yun-Chul Hong, Jong-Han Leem, Joon Sakong, Su Young Kim, Chul Gab Lee, Dongmug Kang, Hyung-Do Choi, Nam Kim

**Affiliations:** 1 Department of Medicine, Dankook University College of Medicine, Cheonan, Korea; 2 Department of Preventive Medicine, Dankook University College of Medicine, Cheonan, Korea; 3 Environmental Health Center, Dankook University Medical Center, Cheonan, Korea; 4 Department of Preventive Medicine, Seoul National University College of Medicine, Seoul, Korea; 5 Department of Occupational and Environmental Medicine, Inha University College of Medicine, Incheon, Korea; 6 Department of Preventive Medicine, Yeungnam University College of Medicine, Daegu, Korea; 7 Department of Preventive Medicine, Cheju National University College of Medicine, Jeju, Korea; 8 Department of Occupational Medicine, Chosun University School of Medicine, Gwangju, Korea; 9 Department of Occupational Medicine, Busan National University School of Medicine, Busan, Korea; 10 Radio Technology Research Department, Electronics and Telecommunication Research Institute, Daejeon, Korea; 11 School of Information and Communication Engineering, Chungbuk National University College of Electrical and Computer Engineering, Cheongju, Korea; The University of Queensland, Australia

## Abstract

**Background:**

Concerns have developed for the possible negative health effects of radiofrequency electromagnetic field (RF-EMF) exposure to children’s brains. The purpose of this longitudinal study was to investigate the association between mobile phone use and symptoms of Attention Deficit Hyperactivity Disorder (ADHD) considering the modifying effect of lead exposure.

**Methods:**

A total of 2,422 children at 27 elementary schools in 10 Korean cities were examined and followed up 2 years later. Parents or guardians were administered a questionnaire including the Korean version of the ADHD rating scale and questions about mobile phone use, as well as socio-demographic factors. The ADHD symptom risk for mobile phone use was estimated at two time points using logistic regression and combined over 2 years using the generalized estimating equation model with repeatedly measured variables of mobile phone use, blood lead, and ADHD symptoms, adjusted for covariates.

**Results:**

The ADHD symptom risk associated with mobile phone use for voice calls but the association was limited to children exposed to relatively high lead.

**Conclusions:**

The results suggest that simultaneous exposure to lead and RF from mobile phone use was associated with increased ADHD symptom risk, although possible reverse causality could not be ruled out.

## Introduction

The 21^st^ century is undoubtedly the era of mobile phone communications with five billion mobile phone subscribers worldwide reported at the end of 2009 [1]. A rapidly increasing number of children and adolescents use mobile phones [2] because mobile phones are used not only to keep in touch with relatives and friends but are also used as a platform for expressing one’s identity and as a source of entertainment [3]. Due to the popularity of mobile phones, exposure to radiofrequency electromagnetic fields (RF EMF) from mobile phones has become unavoidable. As negative health effects of RF EMF from mobile phone use, not only the possible carcinogenic effect of RF EMF on human brain, as one of group-2B agents in IARC’s carcinogen classification [4], but also non-malignant neurotoxic effects on developing brain, i.e., neurocognitive or behavioral effects, have been suggested [5], [6].

If absorption of EMF energy proves to have detrimental effects on the brain, the sensitive developing brain of children might be particularly vulnerable [7]. Studies have shown cognitive impairment in rats related to RF EMF exposure from mobile phones [8] and hyperactive and impaired memory in mice exposed to mobile phone RF during fetal period [9]. Increased behavioral problems including hyperactivity and conduct problems in children with perinatal exposure to mobile phones have also been reported [5], [6], but no studies have shown an association between prenatal exposure to mobile phones and neurodevelopmental delays in younger children [10], [11].

The neurotoxicity of lead includes demyelination or hypomyelination of neurons, death of brain cells through apoptosis and excitotoxicity, and disruptive effects on the dopaminergic system [12]. Children exposed to relatively low levels of lead have inattention [13], cognitive loss [14] and may develop Attention Deficit Hyperactivity Disorder (ADHD) [15], [16].

The blood brain barrier (BBB) is an intricate hydrophobic barrier formed by vascular endothelium of cerebral capillaries with tight junctions between these endothelium cells and plays a pivotal role maintaining homeostasis of the central nervous system by protecting the brain from potentially harmful substances in the blood through strict control of selective diffusion [17]–[21]. A debate is ongoing over the effect of RF EMF exposure on BBB permeability. Numerous studies have reported no changes in BBB permeability after exposure to RF EMF [17], [22], [23]; changes in permeability (if present) have been attributed to an increase in temperature-induced blood flow [17], [20], [24], [25]. However, other studies [19], [26]–[29] have consistently reported increased BBB permeability after exposure to EMF.

ADHD is a behavioral syndrome that is usually diagnosed early in childhood and is characterized by impaired behavioral inhibition, inattention, and physical restlessness [30], [31]. Despite the heterogeneous nature of ADHD, dysfunction of the dopamine modulated frontostriatal circuit of the brain is regarded as one of the core mechanisms associated with ADHD [30]–[32].

The goal of this study was to examine an association between mobile phone use and risk for ADHD symptoms, considering a possible combined effect with lead exposure.

## Materials and Methods

### Ethics Statement

This study was approved by the Institutional Review Boards of the Dankook University Hospital and Asan Medical Center. Written informed consent was obtained from all participant’s parents or guardians after they were fully informed of the study details.

### Subjects

**Figure 1:**
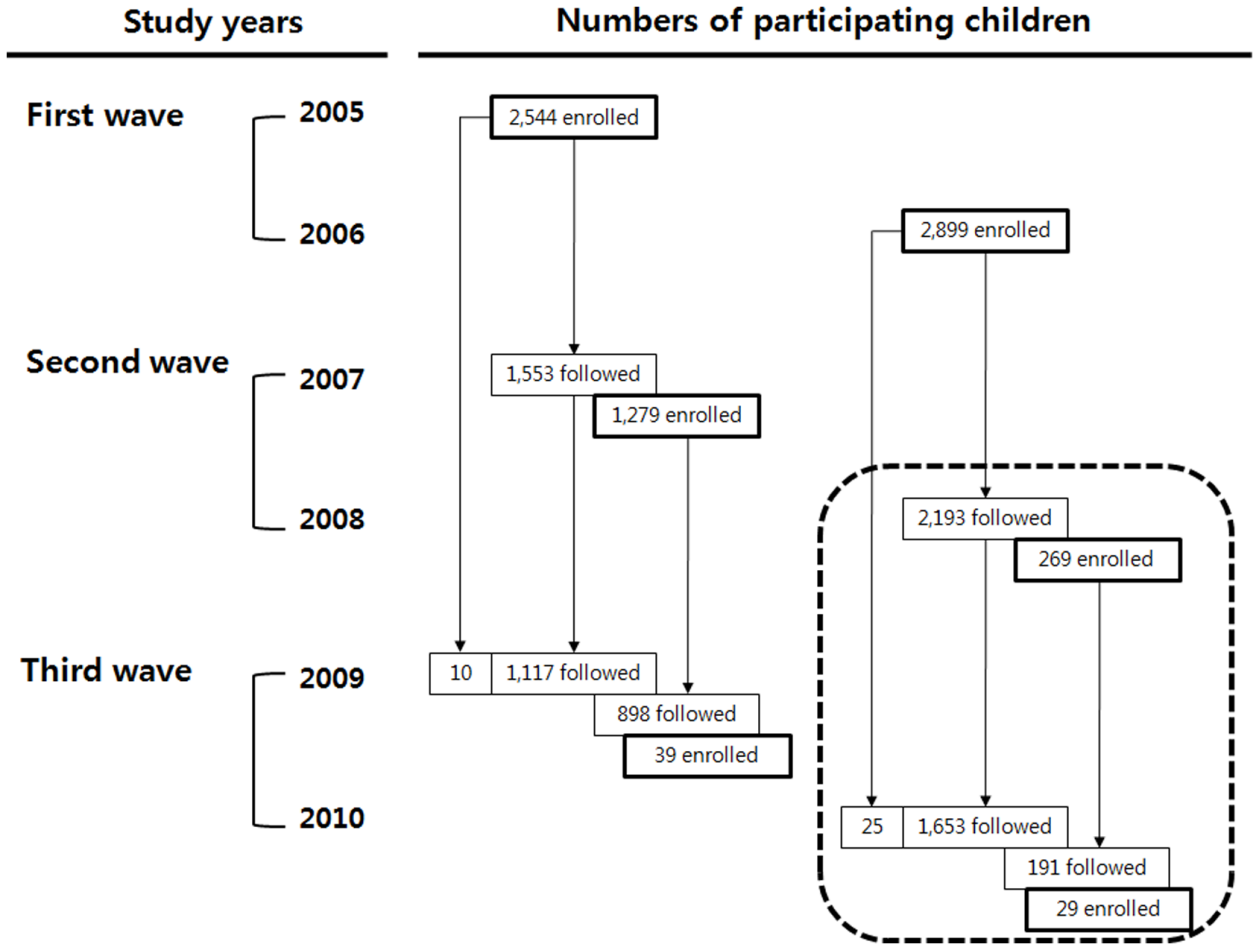
Number of children participating in the CHEER study by survey years. Of 2,516 children at baseline in 2008 and 2010 shown as the dotted lined box, 2,422 were included after excluding children with incomplete questionnaire responses on mobile phone use or a lack of blood lead measurements in 2008 and 2010.

The Children’s Health and Environment Research (CHEER) study was performed to investigate environmental factors associated with health outcomes of school-age children at 27 elementary schools in 10 cities in Korea from 2005 to 2010. CHEER recruited mostly first-grade students from elementary schools in 2005 and 2006 and followed them biennially to 2009 and 2010. A detailed description of the purpose and scope of CHEER has been reported elsewhere [16]. Parents or guardians of a participating child were asked to complete a questionnaire containing questions concerning demographics, socioeconomic status, family environment, symptoms of allergic diseases, and medical and family history. All participating children underwent physical examinations and clinical tests. Among a baseline cohort of 7,059 children in CHEER, 2,516 children whose parents or guardians responded to the questionnaire about children’s mobile phone use, which was administered initially in 2008, were eligible for the present study. A total of 2,422 children were analyzed after excluding children with incomplete information on the questionnaire or an absence of blood lead levels (Fig. 1). The 2008 survey was primarily aimed to follow-up children who were enrolled in the CHEER study in 2006 (average participation rate, 85%) and the follow-up rate in 2008 was 75.6 % (2,193 of 2,899). The newly enrolled 269 children in 2008 from the study schools were not asked to participate but volunteered.

### Information on Mobile Phone Use

Information on mobile phone use was obtained from the questionnaires administered to parents or guardians in 2008 and 2010, i.e., the ownership of a mobile phone by children (no, yes), mobile phone accessibility (do not own or use a mobile phone, do not own a mobile phone but use others own a mobile phone), age when first owned a mobile phone (≥11, 10, 9, ≤ 8 years old), monthly mobile phone bill (<20, 20–24, ≥ 25 10^3^ KRW, 1 USD equals approximately 1,300 KRW as of 2008, 12, 22), average time of mobile phone use per day (no use, <30, ≥ 30 minutes), number of received (outgoing) calls per day (no use, 1–2, ≥ 3), average time spent per voice call (no use, <30 seconds, 30 seconds to <1 minute, ≥ 1 minute), number of received (sent) text messages a day (no use, 1–2, ≥ 3), average time spent playing games on a mobile phone per day (no use, <3, ≥ 3 minutes ), and use of the Internet on a mobile phone (no, yes).

We created a variable of cumulative time spent for voice calls using their own mobile phone (0, <30, 30–69, ≥ 70 hours) using four variables: number of received calls per day plus the number of outgoing calls per day (input 0, 1.5, and 4.5 calls for each category, consecutively) and then multiplied by the average time spent per voice call (input 0, 15, 45, and 105 seconds for each category, consecutively) and duration of mobile phone owned (attained age minus age for first mobile phone). Children who did not own but used someone else’s mobile phone were excluded from creating this variable because of a lack of information for duration of mobile phone use among them.

### ADHD

The Korean version of the ADHD rating scale (K-ARS) was administered to parents or guardians to assess symptoms of the children [33] in 2008 and 2010. A rating of 0–3 (depending on symptom severity) was used for each of the 18 questions and the results were summed. Total scores of ≥19 were regarded as positive for ADHD symptoms.

### Blood Lead Measurements

To measure lead concentrations in blood, 3–5 ml of whole blood was drawn from each child using a syringe and sealed in a heparin containing tube. The lead levels of children’s blood were determined by atomic absorption spectrophotometry (Spectral AA-800®, Varian Inc., Sydney, Australia) at a commercial laboratory. The coefficient of variation for the blood lead levels was 4.9%. Blood lead was measured in 2008 and 2010.

### Other Factors

Information on basic demographic variables and prenatal risk factors for ADHD symptoms was obtained from the questionnaire survey, i.e., residential area (urban, industrial, suburban), parental marital status (couple, single), household income (<2000, 2000–3999, ≥4000 10^3^ KRW per month), number of siblings (0, 1, 2, 3 or more), maternal smoking during pregnancy (no, yes), child’s history of neuropsychiatric disease (no, yes), and parental history of neuropsychiatric disease (no, yes). These variables were all treated as time-independent in the analysis using the generalized estimating equation (GEE) model.

### Statistical Analysis

To examine the association between mobile phone use and ADHD symptoms considering blood lead levels in children, logistic regression analyses were performed at each time point of 2008 and 2010 and the repeatedly measured data from October–November 2008 and October–November 2010 were analyzed using the GEE. The adjusted odds ratios of positive ADHD symptoms and 95% confidence intervals associated with mobile phone use (as time-varying variables in the GEE model) were estimated, adjusted for age, gender, number of siblings, residential area, household income, maternal smoking during pregnancy, child’s history of neuropsychiatric illness, parental marital status, parental history of neuropsychiatric disease (as time-independent covariates in the GEE model), and blood lead levels (as time-varying variables in the GEE model). The p-value for the trend was calculated using the ordinal scale of the mobile phone use variable in the corresponding model.

Analyses were repeated after stratification by blood lead level (cut-off of 2.35 µg/dl; upper 75% point of the distribution of the higher one of two blood lead values measured in 2008 and 2010). The p-values for multiplicative interactions were estimated using the corresponding models including the interaction term to examine the modifying effect of blood lead on the association between mobile phone use and ADHD symptom. The analyses were performed again using the model simultaneously adjusted for three mobile phone use variables, i.e., age at first ownership of a mobile phone, average time spent per voice call, and average time spent playing games. All significance tests were two sided at the 0.05 level, and the analyses were conducted using SAS version 9.1 (SAS Institute, Cary, NC, USA).

## Results

### Study Population and Mobile Phone Use Pattern (Longitudinal Study)

The prevalence of ADHD symptoms in the present study was 10.4% in 2008 and 8.4% in 2010 (Table 1). Ownership of a mobile phone increased almost three times and the cumulative time spent for voice call use increased almost two times over 2 years. The geometric mean (geometric standard deviation) level of blood lead decreased slightly for the 2 years. Very few mothers smoked cigarettes during pregnancy and 1.4% of parents had a history of neuropsychiatric disease (Table 1).

**Table 1:** Characteristics and Mobile Phone Use Patterns of Children in 2008 to 2010, Korea, the CHEER study.

Time-varying Characteristics				
Year of survey	2008 (N = 2,281)		2010 (N = 1,757)	
Age (years), Mean (SD)	8.94	(0.74)	10.58	(0.76)
Blood lead level (µg/dl), GM (GSD)	1.64	(1.54)	1.60	(1.53)
ADHD symptom positive, N (%)	238	(10.4)	147	(8.4)
Ownership of mobile phone (yes), N (%)	518	(22.7)	1,133	(64.5)
Cumulative spent time for voice call (hours)*, Mean (SD)	1.36	(0.65)	2.33	(1.05)
**General Characteristics at Enrollment (N = 2,422)**	**N**	**(%)**		
Gender				
Male	1,236	(51.0)		
Female	1,186	(49.0)		
Residential area				
Urban (5 schools)	814	(33.6)		
Industrial (10 schools)	902	(37.2)		
Suburban (12 schools)	706	(29.1)		
Household income (10^3^ KRW/month)				
<2,000	579	(23.9)		
2,000–<4,000	1,190	(49.1)		
≥4,000	494	(20.4)		
Unknown	159	(6.6)		
Number of siblings				
0	353	(14.6)		
1	1,163	(48.0)		
2	446	(18.4)		
3 or more	76	(3.1)		
Unknown	384	(15.9)		
Parental marital status				
Couple	2,007	(82.9)		
Single	315	(13.0)		
Unknown	100	(4.1)		
Maternal smoking during pregnancy				
No	1,948	(80.4)		
Yes	13	(0.5)		
Unknown	461	(19.0)		
Child's history of neuropsychiatric disease				
No	1,767	(73.0)		
Yes	29	(0.8)		
Unknown	635	(26.2)		
Parental history of neuropsychiatric disease				
No	2,387	(98.6)		
Yes	35	(1.4)		

CHEER, Children’s Health and Environmental Health Research, ADHD, Attention Deficit Hyperactivity Disorder. 1 USD equals approximately 1,084.5 KRW as of 8/9/2011.

* Among children who owned a mobile phone.

### Adjusted for Lead Exposure

Table 2 shows the results of analyses adjusted for several covariates and blood lead on the association between mobile phone use and ADHD symptom for each time point and combined with data of two time points. Ownership of a mobile phone, age at first ownership of a mobile phone, and text message mobile phone use was not associated with ADHD symptoms. Voice-call use variables (number of outgoing calls per day, average time spent per voice call, and cumulative time spent for voice calls) showed increased risks for ADHD symptoms according to increasing mobile phone exposure. In particular, statistical significance was observed for ADHD symptom risk and dose-response trends for number of outgoing calls per day and average time spent per voice call. Mobile phone use for playing games or internet use was significantly associated with ADHD symptoms. Compared to the unadjusted models, the models adjusted for several covariates and blood lead level showed a higher risk for ADHD symptoms in the voice-call use variables, whereas the estimated risk for ADHD symptoms did not change substantially between models for other kinds of mobile phone use, i.e., games or internet. A significant confounding effect of blood lead was observed between ADHD symptoms and voice call use but not other kinds of mobile phone use.

**Table 2:** Association between Mobile Phone Use and ADHD in Children in 2008 and 2010, Korea, the CHEER study.

Mobile phone use variables	Logistic Regression Analysis						Generalized Estimating Equation Analysis						
	2008 (N = 2,281)			2010 (N = 1,757)			2008–2010 (N = 2,422)						
	OR1	OR2	95% CI	OR1	OR2	95% CI	OR1		OR2			95% CI	
Ownership of mobile phone													
No	1	1	(ref)	1	1	(ref)	1		1			(ref)	
Yes	0.78	0.93	(0.64, 1.33)	0.65	0.74	(0.50 1.10)	0.78		0.88			(0.69, 1.14)	
Age at first own of mobile phone*													
11 or more years	1	1	(ref)	1	1	(ref)	1		1			(ref)	
10 years	0.83	1.08	(0.17, 7.01)	1.18	1.22	(0.70, 2.12)	1.26		1.16			(0.68, 1.97)	
9 years	0.76	0.93	(0.18, 4.72)	0.95	1.13	(0.59, 2.20)	1.01		1.09			(0.57, 2.07)	
8 or less years	0.46	0.77	(0.13, 4.61)	0.62	0.90	(0.25, 3.19)	0.65		0.90			(0.25, 3.19)	
*p-*trend	*0.25*	*0.93*		*0.23*	*0.84*		*0.31*		*0.89*				
Number of sent text messages per day													
No use	1	1	(ref)	1	1	(ref)	1		1			(ref)	
1–2	0.63	0.90	(0.51, 1.57)	0.72	0.82	(0.48, 1.40)	0.76		0.90			(0.63, 1.29)	
3 or more	0.91	1.18	(0.70, 1.98)	0.59	0.73	(0.47, 1.12)	0.80		1.00			(0.73, 1.38)	
*p-*trend	*0.32*	*0.68*		*0.01*	*0.14*		*0.08*		*0.17*				
Number of outgoing calls per day													
No use	1	1	(ref)	1	1	(ref)	1		1			(ref)	
1–2	1.02	1.30	(0.90, 1.87)	1.11	0.99	(0.60, 1.65)	0.98		1.20			(0.90, 1.59)	
3 or more	0.81	1.11	(0.65, 1.91)	1.08	1.46	(0.95, 2.25)	1.09		1.39			(1.02, 1.88)	
*p-*trend	*0.54*	*0.32*		*0.67*	*0.08*		*0.62*		*0.03*				
Average time spent per voice call													
No use	1	1	(ref)	1	1	(ref)	1		1			(ref)	
<30 seconds	0.75	0.92	(0.64, 1.31)	0.92	1.17	(0.52, 2.63)	0.81		0.96			(0.70, 1.31)	
30 seconds-<1 minute	0.93	1.28	(0.87, 1.91)	1.15	1.49	(0.70, 3.14)	1.01		1.27			(0.93, 1.75)	
1 or more minute	1.11	1.32	(0.79, 2.21)	1.39	1.91	(0.91, 4.01)	1.23		1.50			(1.06, 2.14)	
*p-*trend	*0.89*	*0.15*		*0.11*	*0.03*		*0.14*		*0.01*				
Cumulative time spent for voice call*													
0	1	1	(ref)	1	1	(ref)	1		1			(ref)	
<30 hours	0.80	1.01	(0.65, 1.57)	0.59	0.71	(0.41, 1.24)	0.80		0.92			(0.66, 1.28)	
30–<70 hours	0.99	1.29	(0.47, 3.55)	0.78	1.09	(0.55, 2.15)	0.96		1.29			(0.76, 2.20)	
70 or more hours	0.87	1.42	(0.39, 5.10)	0.86	1.15	(0.61, 2.17)	0.98		1.33			(0.78, 2.25)	
*p-*trend	*0.46*	*0.58*		*0.87*	*0.40*		*0.81*		*0.27*				
Average time spent for playing games on mobile phone per day													
No use	1	1	(ref)	1	1	(ref)	1	1		(ref)			
1–2	1.65	1.54	(0.88, 2.69)	0.86	0.83	(0.45, 1.53)	1.17	1.12		(0.75, 1.68)			
3 minutes or more	2.05	1.94	(1.30, 2.89)	1.81	1.66	(1.00, 2.50)	1.88	1.81		(1.36, 2.40)			
*p-*trend	*<.0001*	*0.04*		*0.01*	*0.35*		*<.0001*	*<.0001*					
Use of internet on mobile phone													
No	1	1	(ref)	1	1	(ref)	1	1		(ref)			
Yes	2.99	2.56	(1.05, 6.25)	1.44	1.38	(0.07, 2.72)	1.82	1.76		(0.02, 1.55)			

CHEER, Children’s Health and Environmental Health Research; ADHD, Attention Deficit Hyperactivity Disorder.

Odds ratio (OR)1 estimated using simple logistic regression (in 2008 or 2010) or unadjusted generalized estimating equation analysis (2008–2010).

OR2 in 2008 or 2010 estimated using multiple logistic regression analysis for each time point after adjusting for age, gender, number of siblings, area, household income, maternal smoking during pregnancy, child’s history of neuropsychiatric illness, parental history of neuropsychiatric illness, parental marital status, and blood lead level.

OR2 in 2008 and 2010 estimated using a generalized estimating equation analysis for repeated measure at two time points after adjusting for age, gender, number of siblings, area, household income, maternal smoking during pregnancy, child’s history of neuropsychiatric illness, parental history of neuropsychiatric illness, and parental marital status as time-independent covariates and blood lead levels as time-varying covariates.

* Among children who owned their mobile phone.

### Stratified by Lead Exposure

In the stratified analysis by blood lead level (Table 3), the variables of voice call use (number of outgoing calls per day, average time spent per voice call, and cumulative time spent for voice calls) showed significant associations and/or trends only in children with a high blood lead level. In contrast, use of a mobile phone for playing games or using the internet was significantly associated with ADHD symptoms only in children with a low blood lead level. Mobile phone ownership, age at first ownership, and text message use were not significantly associated with low or high blood lead levels in either group. Multiplicative interaction tests between mobile phone exposure and blood lead in association with ADHD symptoms were significant or borderline significant for the variables of ownership, age at first ownership of a mobile phone, and the number of outgoing calls per day, and the risk of ADHD symptoms was higher in the high lead group than that in the low lead group.

**Table 3:** Association Between Mobile Phone Use and ADHD in Children Stratified by the Blood Lead Level in 2008 and 2010, Korea, the CHEER study.

Blood Lead Level	Low (<2.35 µg/dl)			High (≥2.35 µg/dl)		P for interaction	
	(N = 1,788, ADHD = 180)			(N = 600, ADHD = 69)			
	OR		(95% CI)	OR	(95% CI)		
Ownership of mobile phone							
No	1	(ref)		1	(ref)		
Yes	0.69	(0.51, 0.94)		1.35	(0.75, 2.43)	*0.06*	
Age at first own of mobile phone*							
11 or more years	1	(ref)		1	(ref)		
10 years	0.88	(0.48, 1.59)		2.90	(0.74, 11.34)		
9 years	0.91	(0.45, 1.82)		1.08	(0.17, 6.89)		
8 or less years	0.67	(0.16, 2.79)		1.67	(0.14, 20.33)		*0.01*
*p-*trend	*0.65*			*0.15*			
Number of sent text messages per day							
No use	1	(ref)		1	(ref)		
1–2	0.73	(0.47, 1.13)		1.36	(0.60, 3.10)		
3 or more	0.78	(0.53, 1.16)		1.45	(0.07, 3.00)		*0.16*
*p-*trend	*0.16*			*0.29*			
Number of outgoing calls per day							
No use	1	(ref)		1	(ref)		
1–2	1.00	(0.70, 1.43)		1.67	(0.87, 3.18)		
3 or more	1.13	(0.78, 1.62)		2.46	(1.21, 4.97)		*0.06*
*p-*trend	*0.57*			*0.01*			
Average time spent per voice call							
No use	1	(ref)		1	(ref)		
<30 seconds	0.79	(0.54, 1.16)		1.16	(0.57, 2.36)		
30 seconds-<1 minute	1.08	(0.74, 1.57)		1.86	(0.90, 3.84)		
1 or more minute	1.25	(0.82, 1.91)		2.82	(1.29, 6.17)	*0.10*	
*p-*trend	*0.19*			*0.01*			
Cumulative time spent for voice call*							
0	1	(ref)		1	(ref)		
<30 hours	0.71	(0.48, 1.06)		1.35	(0.66, 2.74)		
30–<70 hours	0.91	(0.47, 1.79)		2.75	(0.90, 8.43)		
70 or more hours	1.15	(0.64, 2.07)		2.50	(0.77, 8.01)	*0.47*	
*p-*trend	*0.94*			*0.07*			
Average time spent for playing games on mobile phone per day							
No use	1	(ref)		1	(ref)		
1–2	1.19	(0.75, 1.88)		0.99	(0.37, 2.67)		
3 minutes or more	1.69	(1.20, 2.39)		1.60	(0.84, 3.07)	*1.00*	
*p-*trend	*0.003*			*0.18*			
Use of internet on mobile phone							
No	1	(ref)		1	(ref)		
Yes	1.92	(1.09, 3.38)		0.57	(0.13, 2.38)	*0.16*	

CHEER, Children’s Health and Environmental Health Research, ADHD, Attention Deficit Hyperactivity Disorder.

Odds ratios and 95% confidence intervals were estimated using the generalized estimating equation model adjusted for age, gender, number of siblings, area, household income, maternal smoking during pregnancy, child’s history of neuropsychiatric illness, parental marital status, and parental history of neuropsychiatric disease as time-independent covariates.

p-trend calculated using the ordinal scale of the variable in the corresponding model.

The cut-off point of the high and low groups was the upper 25 percentile of the distribution of the higher between two blood lead levels in 2008 and 2010.

p for multiplicative interaction between blood lead level (high vs. low) and time-varying variables of mobile phone use as a continuous scale.

* Among children who owned a mobile phone.

Stratified analyses were performed repeatedly after including continuous scale of blood lead levels in the corresponding model to examine a possible residual confounding effect of blood lead, but the results did not change (data not shown).

### Simultaneously Adjusted for Different Mobile Phone use Variables

The repeated analyses with full models including three mobile phone use variables simultaneously (age at first ownership of a mobile phone, average time spent per voice call, and average time playing games on a mobile phone per day) to adjust for the variable effects of each mobile phone variable on ADHD symptoms, the results showed a similar pattern with those of the single models in Table 3. However, an increased risk for ADHD symptoms was observed for voice call use in the high blood lead group (Table 4).

**Table 4:** Simultaneous Model of Mobile Phone Use Variables Associated with ADHD in Children Stratified by the Blood Lead Level, 2008–2010, Korea, the CHEER study.

	Blood Lead Level	Low (<2.35 µg/dl)		High (≥2.35 µg/dl)		P for interaction
		(N = 1,788, ADHD = 180)		(N = 600, ADHD = 69)		
		OR	(95% CI)	OR	(95% CI)	
Age at first own of mobile phone*						
11 or more years		1	(ref)	1	(ref)	
10 years		0.81	(0.44, 1.49)	3.37	(0.79, 14.35)	
9 years		0.96	(0.48, 1.9)	1.63	(0.23, 11.56)	
8 years		0.71	(0.17, 2.97)	1.80	(0.11, 29.96)	*0.04*
*p-*trend		*0.69*		*0.51*		
Average time spent per voice call						
<30 seconds		1	(ref)	1	(ref)	
30 seconds-<1 minute		1.04	(0.56, 1.91)	5.66	(1.31, 24.51)	
1 or more minute		1.28	(0.69, 2.38)	7.20	(1.37, 37.91)	*0.40*
*p-*trend		*0.41*		*0.02*		
Average time of playing games on mobile phone per day						
No use		1	(ref)	1	(ref)	
1–2		1.02	(0.49, 2.12)	1.62	(0.43, 6.17)	
3 or more		1.42	(0.86, 2.34)	2.46	(0.95, 6.42)	*0.34*
*p-*trend		*0.19*		*0.06*		

CHEER, Children’s Health and Environmental Health Research, ADHD, Attention Deficit Hyperactivity Disorder.

Odds ratios and 95% confidence intervals estimated using the generalized estimating equation model including three mobile phone use variables and simultaneously adjusted for age, gender, number of siblings, area, household income, maternal smoking during pregnancy, child’s history of neuropsychiatric illness, and parental marital status as time-independent covariates.

p-trend calculated using the ordinal scale of the variable in the corresponding model.

The cut-off point of the high and low groups was the upper 25 percentile of the distribution of the higher levels between two blood lead levels in 2008 and 2010.

p for multiplicative interaction between blood lead level (high vs. low) and time-varying variables of mobile phone use as a continuous scale.

* Among children who owned a mobile phone.

## Discussion

This study showed that voice call mobile phone use was associated with increased ADHD symptom risk in a dose-response manner, but only in children with a high blood lead level. However, playing games on a mobile phone was associated with ADHD symptoms in a dose-response manner regardless of blood lead level and was statistically significant in children with a low blood lead level.

### Interpretation

The voice call variables represented RF exposure to the head rather than other parts of the body because a mobile phone is used near a child’s head, whereas the other variables did not necessarily reflect RF exposure to the head. The finding that voice call mobile phone use was associated with ADHD symptoms supports the hypothesis that RF exposure to children’s heads from mobile phone use increases their vulnerability to lead exposure and ADHD, i.e., possible modifying effects of blood lead on the association between RF exposure from mobile phone use and ADHD symptoms. In contrast, the increased risk in children who spent more time playing games on a mobile phone suggested that such behavior might be as a consequence of ADHD, i.e., reverse causality, or might be one of the risk factors for ADHD-like symptoms [34] rather than the effect of RF exposure to the brain.

Recent studies show that the cognitive function of school-age children is affected by mobile phone use [35], [36], but the results were interpreted as behavioral learning effects from frequent use of a mobile phone rather than the effect of RF exposure by using a mobile phone. However, the behavior of children and adolescents is associated with RF electromagnetic network exposure when measured directly for 24 hours [37]. Thus further prospective studies are needed to confirm the relationship between RF exposure from mobile phone use and behavioral problems including ADHD symptoms in children. The present study used prospectively and repeatedly measured data about mobile phone use, blood lead, and prevalent ADHD symptoms over time. However, the causal time direction from RF exposure to the effects could not be validated in the present study because the ADHD symptoms were not newly developed during the 2 year study period, i.e., mobile phone use might not be an initiating factor for ADHD symptoms and the possibility of reverse causality still remained.

**Figure 2:**
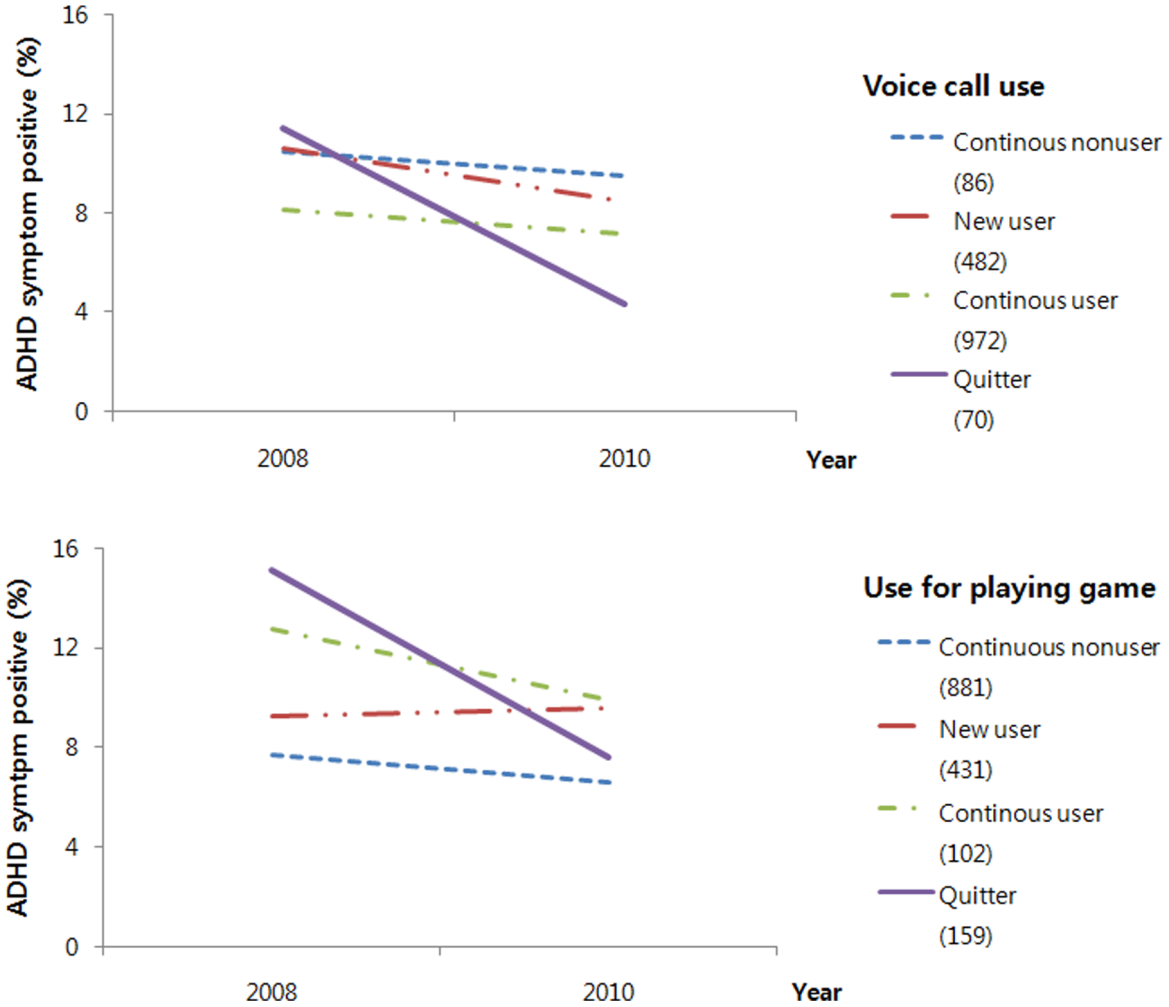
Changes in ADHD symptoms according to the change in mobile phone use for voice calls and playing game over 2 years. Numbers in parentheses are the number of subjects in the corresponding group.

Mobile phone use (voice call use with simultaneous exposure to lead and mobile phone use to play games independently with lead exposure) may aggravate or sustain the ADHD symptoms. In the present study population, the decreasing rate of ADHD symptom prevalence for 2 years was much higher among children who quit mobile phone use compared to the average decreasing rate (−2.0%) in all children: −7.1 and −7.5% in quitters for voice call use and mobile phone use for playing games, respectively (Fig. 2). Therefore, preventing the use of mobile phones in children may be one measure to keep children from developing ADHD symptoms regardless of the possible roles of mobile phone use in ADHD symptoms, i.e., whether potentiating the effect of lead exposure due to RF exposure and voice calls or behavioral aggravation due to high rates of playing games on a mobile phone.

Lead affects heme synthesis and, thus, has an adverse effect on mitochondria that use heme-containing enzymes. This causes significant damage to the BBB that possesses abundant mitochondria and requires a copious supply of ATP [38]. Breakdown of the BBB increases the permeability of lead flowing in the bloodstream and brain parenchyma. Furthermore, lead passes through the BBB through a Ca-ATPase pump [12].

The possible effect of RF EMF’s on the BBB by increasing permeability has been inconsistently reported [39]. In addition, increasing blood flow in the brain induced by thermal effects of EMF continues to be debated with positive [40], [41] or negative effects [42], [43]. Furthermore, meta-analyses of human experimental studies show that EMFs emitted by mobile phones have a small impact on human attention and working memory [44], but no effect on human cognitive performance [45] or cognitive and psychomotor function [46].

An epidemiological study showed a relationship between exposure and health outcome and did not necessarily consider the usually long causal chain and molecular or biological mechanisms in the pathway from exposure to health outcome [47]. Our results suggest that exposure to RF associates with increased ADHD symptom risk with simultaneous exposure to lead, and that RF exposure alone may have a weak or no effect on ADHD symptoms, i.e., a combined or cooperative toxic action of RF and lead on the developing brain. Future studies are needed to confirm this hypothetical mechanism.

### Strength and Limitations

This longitudinal study is the first to examine the combined effects of RF-EMF from mobile phone use and lead exposure in a large population of children. The interpretation of the results from a longitudinal study is more robust than that from a one time cross-sectional study. Furthermore, the follow-up rate of children over the 2 years was moderately high (73.6%), which minimized possible selection bias.

We repeatedly analyzed the data using continuous scale of ADHD score which was log transformed because of the skewed distribution and found similar tendencies with slightly weakened statistical significances (Tables S1–S3).

The limitations of the present study included the following. The ADHD symptom assessment was conducted using the K-ARS, which is used as a preliminary assessment to screen for ADHD symptoms and not for a clinical diagnosis. Moreover, a difference exists between self-reported mobile phone use and actual use of phones in subjects participating in epidemiological studies [48], due to the inherent weakness of retrospective exposure assessments. A validation study on the reports of mobile phone use from the questionnaire survey, i.e., comparison with a telecompany’s registry of calls, was not performed. A discrepancy may also have occurred between reports of mobile phone use from parents or guardians and children. Other confounding factors that may have had an effect were not considered in the analysis.

### Conclusion

The results showed an increased risk for ADHD symptoms in association with heavier voice call mobile phone use among children exposed to lead. Further prospective studies to repeat these findings including incident ADHD cases and to elucidate the possible biological mechanisms are needed.

## Supporting Information

Table S1 **Association between Mobile Phone Use and ADHD in Children in 2008 and 2010, Korea, the CHEER study.** CHEER, Children’s Health and Environmental Health Research; ADHD, Attention Deficit Hyperactivity Disorder. Crude % increase of ADHD score and 95% confidence intervals estimated using simple linear regression (in 2008 or 2010) or unadjusted generalized estimating equation analysis (2008–2010). Adjusted % increase of ADHD score and 95% confidence intervals in 2008 or 2010 estimated using multiple linear regression analysis for each time point after adjusting for age, gender, number of siblings, area, household income, maternal smoking during pregnancy, child’s history of neuropsychiatric illness, parental history of neuropsychiatric illness, parental marital status, and blood lead level. Adjusted % increase in 2008 and 2010 estimated using a generalized estimating equation analysis for repeated measure at two time points after adjusting for age, gender, number of siblings, area, household income, maternal smoking during pregnancy, child’s history of neuropsychiatric illness, parental history of neuropsychiatric illness, and parental marital status as time-independent covariates and blood lead levels as time-varying covariates. *Among children who owned their mobile phone.
(DOCX)

Table S2 **Association between Mobile Phone Use and ADHD in Children Stratified by the Blood Lead Level in 2008 and 2010, Korea, the CHEER study.** CHEER, Children’s Health and Environmental Health Research, ADHD, Attention Deficit Hyperactivity Disorder. % increase of ADHD score and 95% confidence intervals were estimated using the generalized estimating equation model adjusted for age, gender, number of siblings, area, household income, maternal smoking during pregnancy, child’s history of neuropsychiatric illness, parental marital status, and parental history of neuropsychiatric disease as time-independent covariates. p-trend calculated using the ordinal scale of the variable in the corresponding model. The cut-off point of the high and low groups was the upper 25 percentile of the distribution of the higher between two blood lead levels in 2008 and 2010. P for multiplicative interaction between blood lead level (high vs. low) and time-varying variables of mobile phone use as a continuous scale. *Among children who owned a mobile phone.
(DOCX)

Table S3 **Simultaneous Model of Mobile Phone Use Variables Associated with ADHD in Children Stratified by the Blood Lead Level, 2008–2010, Korea, the CHEER study.** CHEER, Children’s Health and Environmental Health Research, ADHD, Attention Deficit Hyperactivity Disorder. % increase of ADHD score and 95% confidence intervals estimated using the generalized estimating equation model including three mobile phone use variables and simultaneously adjusted for age, gender, number of siblings, area, household income, maternal smoking during pregnancy, child’s history of neuropsychiatric illness, and parental marital status as time-independent covariates. P-trend calculated using the ordinal scale of the variable in the corresponding model. The cut-off point of the high and low groups was the upper 25 percentile of the distribution of the higher levels between two blood lead levels in 2008 and 2010. P for multiplicative interaction between blood lead level (high vs. low) and time-varying variables of mobile phone use as a continuous scale. *Among children who owned a mobile phone.
(DOCX)
