# Supplementary material for: Mobile Phone Use, Blood Lead Levels, and Attention Deficit Hyperactivity Symptoms in Children: A Longitudinal Study
Source: PLoS One. 2013 Mar 21;8(3):e59742. doi: 10.1371/journal.pone.0059742 (PMC3605379; doi:10.1371/journal.pone.0059742)
Supplement: Table S1 — Association between Mobile Phone Use and ADHD in Children in 2008 and 2010, Korea, the CHEER study. CHEER, Children’s Health and Environmental Health Research; ADHD, Attention Deficit Hyperactivity Disorder. Crude % increase of ADHD score and 95% confidence intervals estimated using simple linear regression (in 2008 or 2010) or unadjusted generalized estimating equation analysis (2008–2010). Adjusted % increase of ADHD score and 95% confidence intervals in 2008 or 2010 estimated using multiple linear regression analysis for each time point after adjusting for age, gender, number of siblings, area, household income, maternal smoking during pregnancy, child’s history of neuropsychiatric illness, parental history of neuropsychiatric illness, parental marital status, and blood lead level. Adjusted % increase in 2008 and 2010 estimated using a generalized estimating equation analysis for repeated measure at two time points after adjusting for age, gender, number of siblings, area, household income, maternal smoking during pregnancy, child’s history of neuropsychiatric illness, parental history of neuropsychiatric illness, and parental marital status as time-independent covariates and blood lead levels as time-varying covariates. *Among children who owned their mobile phone. (DOCX) [file pone.0059742.s001.docx]

Table S1. Association between Mobile Phone Use and ADHD in Children in 2008 and 2010, Korea, the CHEER study

| Mobile phone use variables | Regression analysis | | | | | | Generalize estimating equation | | |
| --- | --- | --- | --- | --- | --- | --- | --- | --- | --- |
|  | 2008 | | | 2010 | | | 2008-2010 | | |
| log(Y) | Crude | Adjusted | | Crude | Adjusted | | Crude | Adjusted | |
|  | % increase | % increase | 95% CI | % increase | % increase | 95% CI | % increase | % increase | 95% CI |
| Ownership of mobile phone |  |  |  |  |  |  |  |  |  |
| No |  |  |  |  |  |  |  |  |  |
| Yes | -11.27 | -2.59 | (-11.8, 6.6) | -18.34 | -10.72 | (-20.2, -1.2) | -12.6 | -6.4 | (-12.8, 0.0) |
| Age at first own of mobile phone* | | | | | | | | | |
| 11 or more years |  |  |  |  |  |  |  |  |  |
| 10 years | 2.98 | 8.31 | (-42.2, 58.8) | 1.13 | 0.94 | (-11.6, 13.5) | 1.3 | 1.3 | (-10.9, 13.4) |
| 9 years | -19.81 | -7.08 | (-52.5, 38.4) | 2.45 | 6.14 | (-8.4, 20.6) | -2.9 | 4.9 | (-9.3, 19.0) |
| 8 or less years | -19.87 | 2.47 | (-44.7, 49.7) | -7.49 | 4.72 | (-20.3, 29.7) | -7.5 | -1.3 | (-22.8, 20.2) |
| *p-*trend | *0.21* | *0.75* |  | *0.92* | *0.32* |  | *0.45* | *0.67* |  |
|  |  |  |  |  |  |  |  |  |  |
| Number of outgoing calls a day | | | | | | | | | |
| No use |  |  |  |  |  |  |  |  |  |
| 1-2 | -1.88 | 6.7 | (-3.0, 16.4) | -15.2 | -7.56 | (-18.9, 3.8) | -7.1 | -1.2 | (-8.3, 5.8) |
| 3 or more | -4.66 | 7.34 | (-6.2, 20.9) | -12.32 | -4.39 | (-14.7, 5.9) | -8.0 | -1.2 | (-8.7, 6.4) |
| *p-*trend | *0.47* | *0.13* |  | *0.02* | *0.44* |  | *0.02* | *0.73* |  |
| Average time spent per voice call | | | | | | | | | |
| No use |  |  |  |  |  |  |  |  |  |
| <30 seconds | -6.3 | 0.4 | (-8.8, 9.5) | -8.7 | -11.2 | (-28.1, 5.7) | -7.7 | -4.4 | (-11.7, 2.9) |
| 30 seconds-<1 minute | 3.9 | 15.0 | (4.5, 25.5) | -0.2 | -0.1 | (-15.8, 15.6) | 1.4 | 6.7 | (-1.1, 14.4) |
| 1 or more minute | -1.0 | 8.7 | (-5.4, 22.8) | -9.3 | -6.3 | (-22.1, 9.6) | -6.1 | -0.7 | (-9.5, 8.2) |
| *p-*trend | *0.70* | *0.01* |  | *0.41* | *0.95* |  | *0.57* | *0.44* |  |
| Cumulative time spent for voice call† | | | | | | | | | |
| 0 |  |  |  |  |  |  |  |  |  |
| <30 hours | -10.1 | -0.6 | (-11.4, 10.2) | -22.2 | -14.2 | (-2.0, -1.4) | -11.7 | -4.8 | (-12.7, 3.0) |
| 30-<70 hours | -7.6 | 4.1 | (-22.3, 30.4) | -15.0 | -2.9 | (-18.9, 13.1) | -5.4 | 5.4 | (-6.8, 17.5) |
| 70 or more hours | 26.0 | 43.3 | (11.9, 74.8) | -14.8 | -1.7 | (-16.8, 13.4) | -5.9 | 6.4 | (-5.8, 18.6) |
| *p-*trend | *0.75* | *0.09* |  | *0.16* | *0.62* |  | *0.26* | *0.25* |  |
|  |  |  |  |  |  |  |  |  |  |
| Number of sent text messages a day | | | | | | | | | |
| No use |  |  |  |  |  |  |  |  |  |
| 1-2 | -7.1 | 8.0 | (-4.9, 20.9) | -22.6 | -16.1 | (-28.8, -3.4) | -11.5 | -4.2 | (-12.7, 4.4) |
| 3 or more | -6.7 | 2.7 | (-10.9, 16.4) | -19.8 | -11.7 | (-21.7, -1.8) | -10.9 | -3.9 | (-11.3, 3.6) |
| *p-*trend | *0.20* | *0.42* |  | *<.0001* | *0.02* |  | *0.00* | *0.27* |  |
| Average time spent for playing games on mobile phone a day | | | | | | | | | |
| No use |  |  |  |  |  |  |  |  |  |
| 1-2 | 3.6 | 2.7 | (-13.2, 18.6) | -2.8 | -1.6 | (-14.8, 11.5) | -2.4 | -2.7 | (-12.3, 7.0) |
| 3 minutes or more | 17.3 | 14.3 | (2.1, 26.5) | 12.7 | 9.1 | (-1.8, 20.0) | 13.2 | 11.3 | (3.8, 18.8) |
| *p-*trend | *0.01* | *0.02* |  | *0.04* | *0.14* |  | *0.00* | *0.01* |  |
| Use of internet on mobile phone | | | | | | | | | |
| No |  |  |  |  |  |  |  |  |  |
| Yes | 28.0 | 21.0 | (-12.0, 54.0) | 7.3 | 8.4 | (-10.1, 2.0) | 7.2 | 6.2 | (-10.4, 22.8) |

CHEER, Children’s Health and Environmental Health Research; ADHD, Attention Deficit Hyperactivity Disorder.

Crude % increase of ADHD score and 95% confidence intervals estimated using simple linear regression (in 2008 or 2010) or unadjusted generalized estimating equation analysis (2008–2010).

Adjusted % increase of ADHD score and 95% confidence intervals in 2008 or 2010 estimated using multiple linear regression analysis for each time point after adjusting for age, gender, number of siblings, area, household income, maternal smoking during pregnancy, child’s history of neuropsychiatric illness, parental history of neuropsychiatric illness, parental marital status, and blood lead level.

Adjusted % increase in 2008 and 2010 estimated using a generalized estimating equation analysis for repeated measure at two time points after adjusting for age, gender, number of siblings, area, household income, maternal smoking during pregnancy, child’s history of neuropsychiatric illness, parental history of neuropsychiatric illness, and parental marital status as time-independent covariates and blood lead levels as time-varying covariates.

*Among children who owned their mobile phone.
