# Supplementary material for: Mobile Phone Use, Blood Lead Levels, and Attention Deficit Hyperactivity Symptoms in Children: A Longitudinal Study
Source: PLoS One. 2013 Mar 21;8(3):e59742. doi: 10.1371/journal.pone.0059742 (PMC3605379; doi:10.1371/journal.pone.0059742)
Supplement: Table S2 — Association between Mobile Phone Use and ADHD in Children Stratified by the Blood Lead Level in 2008 and 2010, Korea, the CHEER study. CHEER, Children’s Health and Environmental Health Research, ADHD, Attention Deficit Hyperactivity Disorder. % increase of ADHD score and 95% confidence intervals were estimated using the generalized estimating equation model adjusted for age, gender, number of siblings, area, household income, maternal smoking during pregnancy, child’s history of neuropsychiatric illness, parental marital status, and parental history of neuropsychiatric disease as time-independent covariates. p-trend calculated using the ordinal scale of the variable in the corresponding model. The cut-off point of the high and low groups was the upper 25 percentile of the distribution of the higher between two blood lead levels in 2008 and 2010. P for multiplicative interaction between blood lead level (high vs. low) and time-varying variables of mobile phone use as a continuous scale. *Among children who owned a mobile phone. (DOCX) [file pone.0059742.s002.docx]

Table S2. Association between Mobile Phone Use and ADHD in Children Stratified by the Blood Lead Level in 2008 and 2010, Korea, the CHEER study

| Blood Lead Level | Low (<2.35 ug/dl) | | High (≥2.35 ug/dl) | |  |
| --- | --- | --- | --- | --- | --- |
|  | (N=1,788, ADHD=180) | | (N=600, ADHD=69) | |  |
| Mobile Use Variables | % increase | (95% CI) | % increase | (95% CI) | p for interaction |
| Ownership of mobile phone |  |  |  |  |  |
| No |  |  |  |  |  |
| Yes | -12.2 | (-19.5, -4.9) | 7.0 | (-5.5, 19.4) | *0.80* |
| Age at first own of mobile phone* | | | | | |
| 11 or more years |  |  |  |  |  |
| 10 years | -3.9 | (-18.3, 10.5) | 17.7 | (-4.4, 39.8) |  |
| 9 years | 6.0 | (-10.1, 22.1) | -3.8 | (-33.8, 26.2) |  |
| 8 or less years | -14.6 | (-42.1, 12.9) | 9.9 | (-26.3, 46.2) | *0.38* |
| *p-*trend | *0.97* |  | *0.94* |  |  |
|  |  |  |  |  |  |
| Number of outgoing calls a day | |  |  |  |  |
| No use |  |  |  |  |  |
| 1-2 | -5.4 | (-13.6, 2.7) | 7.2 | (-6.3, 20.7) |  |
| 3 or more | -5.5 | (-14.2, 3.1) | 9.0 | (-5.4, 23.5) | *0.90* |
| *p-*trend | *0.17* |  | *0.19* |  |  |
| Average time spent per voice call | | | | | |
| No use |  |  |  |  |  |
| <30 seconds | -8.4 | (-16.9, 0) | 2.0 | (-11.8, 15.9) |  |
| 30 seconds-<1 minute | 5.4 | (-3.2, 14) | 6.0 | (-10.4, 22.3) |  |
| 1 or more minute | -2.7 | (-13.1, 7.6) | 3.4 | (-12.8, 19.5) | *0.51* |
| *p-*trend | *0.63* |  | *0.58* |  |  |
| Cumulative time spent for voice call† | | | | | |
| 0 |  |  |  |  |  |
| <30 hours | -9.8 | (-18.7, -0.9) | 8.5 | (-7.8, 24.7) |  |
| 30-<70 hours | -1.7 | (-15.8, 12.4) | 24.9 | (3.1, 46.7) |  |
| 70 or more hours | -1.7 | (-16, 12.7) | 24.2 | (1.9, 46.4) | *0.88* |
| *p-*trend | *0.79* |  | *0.01* |  |  |
|  |  |  |  |  |  |
| Number of sent text messages a day | | | | | |
| No use |  |  |  |  |  |
| 1-2 | -5.4 | (-15.6, 4.7) | 0.4 | (-15.6, 16.5) |  |
| 3 or more | -2.0 | (-11.3, 5.5) | -3.1 | (-17.5, 11.3) | *0.47* |
| *p-*trend | *0.43* |  | *0.69* |  |  |
| Average time spent for playing games on mobile phone a day | | | | | |
| No use |  |  |  |  |  |
| 1-2 | -2.4 | (-13.3, 8.5) | -5.4 | (-25.1, 14.3) |  |
| 3 minutes or more | 9.9 | (1.6, 18.3) | 6.6 | (-8.5, 21.7) | *0.40* |
| *p-*trend | *0.04* |  | *0.50* |  |  |
| Use of internet on mobile phone | | | | | |
| No |  |  |  |  |  |
| Yes | 7.9 | (-11.8, 27.5) | -5.1 | (-34.3, 24.2) | *0.42* |

CHEER, Children’s Health and Environmental Health Research, ADHD, Attention Deficit Hyperactivity Disorder.

% increase of ADHD score and 95% confidence intervals were estimated using the generalized estimating equation model adjusted for age, gender, number of siblings, area, household income, maternal smoking during pregnancy, child’s history of neuropsychiatric illness, parental marital status, and parental history of neuropsychiatric disease as time-independent covariates.

p-trend calculated using the ordinal scale of the variable in the corresponding model.

The cut-off point of the high and low groups was the upper 25 percentile of the distribution of the higher between two blood lead levels in 2008 and 2010.

p for multiplicative interaction between blood lead level (high vs. low) and time-varying variables of mobile phone use as a continuous scale.

*Among children who owned a mobile phone.
