# Supplementary material for: Mobile Phone Use, Blood Lead Levels, and Attention Deficit Hyperactivity Symptoms in Children: A Longitudinal Study
Source: PLoS One. 2013 Mar 21;8(3):e59742. doi: 10.1371/journal.pone.0059742 (PMC3605379; doi:10.1371/journal.pone.0059742)
Supplement: Table S3 — Simultaneous Model of Mobile Phone Use Variables Associated with ADHD in Children Stratified by the Blood Lead Level, 2008–2010, Korea, the CHEER study. CHEER, Children’s Health and Environmental Health Research, ADHD, Attention Deficit Hyperactivity Disorder. % increase of ADHD score and 95% confidence intervals estimated using the generalized estimating equation model including three mobile phone use variables and simultaneously adjusted for age, gender, number of siblings, area, household income, maternal smoking during pregnancy, child’s history of neuropsychiatric illness, and parental marital status as time-independent covariates. P-trend calculated using the ordinal scale of the variable in the corresponding model. The cut-off point of the high and low groups was the upper 25 percentile of the distribution of the higher levels between two blood lead levels in 2008 and 2010. P for multiplicative interaction between blood lead level (high vs. low) and time-varying variables of mobile phone use as a continuous scale. *Among children who owned a mobile phone. (DOCX) [file pone.0059742.s003.docx]

Table S3. Simultaneous Model of Mobile Phone Use Variables Associated with ADHD in Children Stratified by the Blood Lead Level, 2008–2010, Korea, the CHEER study

|  |  | Low (<2.35 ug/dl) | | High (≥2.35 ug/dl) | | P for  Interaction** |
| --- | --- | --- | --- | --- | --- | --- |
|  |  | (N=1,788, ADHD=180) | | (N=600, ADHD=69) | |  |
|  |  | %increase | (95% CI) | %increase | (95% CI) |  |
| Age at first own of mobile phone* | | |  |  |  |  |
|  | 11 or more years |  |  |  |  |  |
|  | 10 years | -4.9 | (-19.4, 9.7) | 15.2 | (-7.5, 37.9) |  |
|  | 9 years | 8.4 | (-7.9, 24.6) | -1.1 | (-31.9, 29.7) |  |
|  | 8 years | -15.6 | (-44.3, 13.1) | 13.4 | (-24.3, 51.1) | *0.17* |
| Average time spent per voice call | | |  |  |  |  |
|  | 0 |  |  |  |  |  |
|  | <30 seconds |  |  |  |  |  |
|  | 30 seconds-<1 minute | 18.3 | (5.6, 31) | 20.6 | (-1.9, 43) |  |
|  | 1 or more minute | 12.8 | (-0.9, 26.4) | 15.6 | (-6.2, 37.5) | *0.47* |
| Average time of playing games on mobile phone a day | | | |  |  |  |
|  | No use |  |  |  |  |  |
|  | 1-2 | -3.4 | (-18.2, 11.5) | -9.5 | (-35.3, 16.4) |  |
|  | 3 or more | 13.8 | (1.5, 26.2) | 11.9 | (-8.5, 32.3) | *0.49* |

CHEER, Children’s Health and Environmental Health Research, ADHD, Attention Deficit Hyperactivity Disorder.

% increase of ADHD score and 95% confidence intervals estimated using the generalized estimating equation model including three mobile phone use variables and simultaneously adjusted for age, gender, number of siblings, area, household income, maternal smoking during pregnancy, child’s history of neuropsychiatric illness, and parental marital status as time-independent covariates.

p-trend calculated using the ordinal scale of the variable in the corresponding model.

The cut-off point of the high and low groups was the upper 25 percentile of the distribution of the higher levels between two blood lead levels in 2008 and 2010.

p for multiplicative interaction between blood lead level (high vs. low) and time-varying variables of mobile phone use as a continuous scale.

*Among children who owned a mobile phone.
